# *ERCC2* gene single-nucleotide polymorphism as a prognostic factor for locally advanced head and neck carcinomas after definitive cisplatin-based radiochemotherapy

**DOI:** 10.1038/s41397-020-0174-1

**Published:** 2020-06-16

**Authors:** Maja Guberina, Ali Sak, Christoph Pöttgen, Ingeborg Tinhofer-Keilholz, Volker Budach, Panagiotis Balermpas, Jens Von der Grün, Claus Michael Rödel, Eleni Gkika, Anca-Ligia Grosu, Amir Abdollahi, Jürgen Debus, Claus Belka, Steffi Pigorsch, Stephani E. Combs, David Mönnich, Daniel Zips, Chiara De-Colle, Stefan Welz, Annett Linge, Fabian Lohaus, Gustavo Baretton, Thomas Gauler, Michael Baumann, Mechthild Krause, Martin Schuler, Agnes Bankfalvi, Benedikt Höing, Stephan Lang, Martin Stuschke

**Affiliations:** 1 Department of Radiotherapy, University Hospital Essen, University of Duisburg-Essen, Essen, Germany; 2 German Cancer Consortium (DKTK), Partner Site Berlin, and German Cancer Research Center (DKFZ), Heidelberg, Germany; 3 Department of Radiooncology and Radiotherapy, Charité—Universitätsmedizin Berlin, Corporate Member of Freie Universität Berlin, Humboldt-Universität zu Berlin, and Berlin Institute of Health, Berlin, Germany; 4 German Cancer Consortium (DKTK), Partner Site Frankfurt, and German Cancer Research Center (DKFZ), Heidelberg, Germany; 5 Department of Radiotherapy and Oncology, Goethe-University Frankfurt, Frankfurt, Germany; 6 Department of Radiation Oncology, Medical Center, Medical Faculty, University of Freiburg, Freiburg, Germany; 7 German Cancer Consortium (DKTK), Partner Site Freiburg, and German Cancer Research Center (DKFZ), Heidelberg, Germany; 8 German Cancer Consortium (DKTK), Partner Site Heidelberg, and German Cancer Research Center (DKFZ), Heidelberg, Germany; 9 Heidelberg Institute of Radiation Oncology (HIRO), National Center for Radiation Research in Oncology (NCRO), University of Heidelberg Medical School, and German Cancer Research Center (DKFZ), Heidelberg, Germany; 10 Department of Radiation Oncology, Heidelberg Ion Therapy Center (HIT), University of Heidelberg Medical School, Heidelberg, Germany; 11 National Center for Tumor Diseases (NCT), Medicine and University Hospital, Technische Universität Dresden, Partner Site Dresden, Germany and German Cancer Research Center (DKFZ), Heidelberg, Germany; 12 Translational Radiation Oncology, University of Heidelberg Medical School, and German Cancer Research Center (DKFZ), Heidelberg, Germany; 13 Clinical Cooperation Unit Radiation Oncology, University of Heidelberg Medical School and German Cancer Research Center (DKF), Heidelberg, Germany; 14 German Cancer Consortium (DKTK), Partner Site Munich, and German Cancer Research Center (DKFZ), Heidelberg, Germany; 15 Department of Radiotherapy and Radiation Oncology, University Hospital, Ludwig-Maximilians-Universität, Munich, Germany; 16 Clinical Cooperation Group Personalized Radiotherapy in Head and Neck Cancer, Helmholtz Zentrum Munich, Neuherberg, Germany; 17 Department of Radiation Oncology, Technische Universität München, Munich, Germany; 18 Department of Radiation Sciences (DRS), Institut für Innovative Radiotherapie (iRT), Helmholtz Zentrum Munich, Neuherberg, Germany; 19 German Cancer Consortium (DKTK), Partner Site Tübingen, and German Cancer Research Center (DKFZ), Heidelberg, Germany; 20 Department of Radiation Oncology, Faculty of Medicine and University Hospital Tübingen, Eberhard Karls Universität Tübingen, Tübingen, Germany; 21 OncoRay—National Center for Radiation Research in Oncology, Faculty of Medicine and University Hospital Carl Gustav Carus, Technische Universität Dresden, Helmholtz-Zentrum Dresden—Rossendorf, Dresden, Germany; 22 Department of Radiotherapy and Radiation Oncology, Faculty of Medicine and University Hospital Carl Gustav Carus, Dresden, Technische Universität Dresden, Dresden, Germany; 23 German Cancer Consortium (DKTK), Partner Site Dresden, and German Cancer Research Center (DKFZ), Heidelberg, Germany; 24 Helmholtz Association/Helmholtz-Zentrum Dresden—Rossendorf (HZDR), Dresden, Germany; 25 German Cancer Research Center (DKFZ), Heidelberg, Germany; 26 Tumor and Normal Tissue Bank, University Cancer Centre (UCC), University Hospital Carl Gustav Carus, Technische Universität Dresden, Dresden, Germany; 27 Helmholtz-Zentrum Dresden—Rossendorf, Institute of Radiooncology—OncoRay, Dresden, Germany; 28 German Cancer Consortium (DKTK), Partner Site University Hospital Essen, and German Cancer Research Center (DKFZ), Essen, Germany; 29 Department of Medical Oncology, West German Cancer Center, University Hospital Essen, University Duisburg-Essen, Hufelandstrasse 55, Essen, Germany; 30 Division of Thoracic Oncology, University Medicine Essen-Ruhrlandklinik, University Duisburg-Essen, Essen, Germany; 31 Institute for Pathology, University Hospital Essen, University of Duisburg-Essen, Essen, Germany; 32 Department of Otorhinolaryngology, University Hospital Essen, University Hospital Duisburg-Essen, Essen, Germany

**Keywords:** Cancer genetics, Prognostic markers

## Abstract

Identifying patients with locally advanced head and neck carcinoma on high risk of recurrence after definitive concurrent radiochemotherapy is of key importance for the selection for consolidation therapy and for individualized treatment intensification. In this multicenter study we analyzed recurrence-associated single-nucleotide polymorphisms (SNPs) in DNA repair genes in tumor DNA from 132 patients with locally advanced head and neck carcinoma (LadHnSCC). Patients were treated with definitive radiotherapy and simultaneous cisplatin-based chemotherapy at six partner sites of the German Cancer Consortium (DKTK) Radiation Oncology Group from 2005 to 2011. For validation, a group of 20 patients was available. Score selection method using proportional hazard analysis and leave-one-out cross-validation were performed to identify markers associated with outcome. The SNPs rs1799793 and rs13181 were associated with survival and the same SNPs and in addition rs17655 with freedom from loco-regional relapse (ffLRR) in the trainings datasets from all patients. The homozygote major rs1799793 genotype at the *ERCC2* gene was associated with better (Hazard ratio (HR): 0.418 (0.234–0.744), *p* = 0.003) and the homozygote minor rs13181 genotype at *ERCC2* with worse survival (HR: 2.074, 95% CI (1.177–3.658), *p* = 0.017) in comparison to the other genotypes. At the ffLRR endpoint, rs1799793 and rs13181 had comparable prognostic value. The rs1799793 and rs13181 genotypes passed the leave-one-out cross-validation procedure and associated with survival and ffLRR in patients with LadHnSCC treated with definitive radiochemotherapy. While findings were confirmed in a small validation dataset, further validation is underway within a prospective biomarker study of the DKTK.

## Introduction

Definitive cisplatin-based radiochemotherapy of locally advanced squamous cell carcinoma of the head and neck achieves 5-year survival rates of about 30–50% in patients with HPV-negative tumors, treated in prospective trials [1, 2]. At such event rates radiation dose–response relationship is often the steepest and correlates positively with higher radiation doses. At higher or lower event rates, larger samples are needed to precisely determine development of classifier for progression free and overall survival [3]. Cisplatin-based radiochemotherapy is one of the standard treatment approaches in locally advanced head and neck carcinoma [4]. Nuclear excision repair pathways are the main mechanism to repair cisplatin–DNA adducts [5] and also mitomycin C induced DNA interstrand cross-links [6]. Single-nucleotide polymorphism (SNP) in nuclear excision repair as well as single or double strand break repair genes have been observed in several retrospective analyses being associated with a prognostic outcome of head and neck cancer patients treated with radiotherapy (RTX) or radiochemotherapy (RT/CTX) at the clinical endpoints for normal tissue toxicity, tumor response, or survival [7–13]. However, none of these SNPs plays any role in clinical routine for treatment selection or prognosis prediction so far.

A multicentre retrospective biomarker study on patients with locally advanced squamous cell head and neck carcinoma treated with definitive RT/CTX was initiated by the partners of the Radiation Oncology Group of the German Cancer Consortium (DKTK-ROG) with a purpose to establish prognostic and/or predictive biomarkers [14, 15]. In the present study, the prognostic value of SNPs in repair proteins relevant for the effectiveness of the combined cisplatin and radiation therapy was analyzed on a cohort of patients homogeneously treated with definitive radiotherapy and concurrent chemotherapy. A separate group of patients receiving cisplatin-based induction chemotherapy in addition to concurrent radiochemotherapy was used for validation.

## Materials and methods

### Study population and treatment

Patients of the DKTK-ROG biomarker study with loco-regionally advanced head and neck squamous cell carcinoma of the oral cavity, oro- and hypopharynx, who were treated with definitive radiotherapy and simultaneous chemotherapy from 2005 to 2011 at six partner sites, were eligible for the present study. This study included 158 patients. The characteristics of the patients were previously described [14]. The following clinical factors describing the extent of disease and general criteria of each patient were obtained before definitive radiochemotherapy: age, gender, lymph node category, p16 expression, tumor site, and the logarithm of the combined gross tumor volume of the primary tumor and involved lymph nodes log(GTV_total_) [14]. Patients without available genomic DNA for translational research centrally prepared in Dresden from formalin-fixed paraffin-embedded (FFPE) specimens of the primary tumor, had to be excluded (*n* = 23). In addition, patients without GTV measurements as a major prognostic clinical factor (*n* = 1) or by missing or unequivocal genotype data from SNP analysis (*n* = 2, sample call rate = 98.5%) were excluded. Patients documented by the DKTK-ROG, who received induction cisplatin-based chemotherapy and definitive radiotherapy with concurrent cisplatin-based chemotherapy during the same time period were eligible as a validation group. Ethical approvals for retrospective analyses of the clinical and biological data were granted by the ethics committees of all DKTK partner sites.

### SNP selection and genotyping assays

Genomic DNA samples extracted from the FFPE-tumor probes were used to genotype eight SNPs localized in six genes. The genomic DNA was extracted from 5 μm thick FFPE sections using the QIAamp DNA FFPE tissue kit (Qiagen, Hilde, Germany). The analyzed genes included the nucleotide excision repair pathways *ERCC2* (*XPD*) (rs1799793, rs13181, rs50871), *ERCC5* (*XPG*) (rs17655), *ERCC1* (rs11615), nonhomologous end-joining repair *XRCC6* (rs2267437) as well as *ATM* (rs4988023), and single strand break repair *XRCC1* (rs25487). The selection of these SNPs is based on a thorough literature search in PubMed performed in mid-2016. Only SNPs with existing data on a prognostic association with outcome of carcinoma after definitive radiochemotherapy and toxicity were included. References that led to the inclusion of the different SNPs were as follows: [8, 11, 16] for rs1799793, [7, 8, 12] for rs13181, [9] for rs4988023, [11, 17] for rs17655, [8] for rs25487, [18] for rs50871, [19] for rs2267437, and [20] for rs11615, respectively.

TaqMan allele discrimination assays were run on the ABI 7700 Sequence Detection System (Applied Biosystems, Rotkreuz, Switzerland) to determine the genotypes which use the TaqMan 5′-nuclease chemistry to amplify and detect specific polymorphisms in purified genomic DNA samples. Each assay enabled genotyping of individuals for an SNP and consists of two sequence-specific primers as well as two TaqMan minor groove binder probes with nonfluorescent quenchers. The probes are labeled with VIC and FAM dyes to detect the Allele 1 and Allele 2 sequences, respectively. Genotyping of SNPs was performed 2–3 times (*n*) for each SNP using the TaqMan allelic discrimination assays (rs50871, C_958480_10 (*n* = 2); rs1799793, C_3145050 (*n* = 2); rs13181, C_3145033_10 (*n* = 2); rs17655, C_1891743_10 (*n* = 3); rs11615, C_2532959_1 (*n* = 2); rs2267437, C_15872242_20 (*n* = 3); rs4988023, C_33307846_10 (*n* = 2); rs25487, C_622564_10 (*n* = 2)) all from Thermo Fisher Scientific, USA, on an ABI Prism 7900 HT Sequence Detection System (Applied Biosystems). The following cycling conditions were used: 10 min at 95 °C, 45 cycles of 95 °C for 15 sec, and 60 °C for 1 min. About 5 ng of each genomic DNA were utilized per polymerase chain reaction in a volume of 5 μl. The analysis was done by using the SDS2.2 software package from Applied Biosystems. The full prognostic genotype information at each SNP locus was classified by two dummy variables rsSNP-1 and rsSNP-2. The rsSNP-1 contrasts the homozygote major allele phenotype against the heterozygote or homozygote minor alternatives, while the rsSNP-2 contrasts the homozygote minor genotype against the two other genotypes. This genetic model free approach also recommended [21] was selected, because there was not enough a priori evidence available to justify a specific genetic model for a given SNP.

### Outcome definition

The first endpoint of this study was overall survival and the second endpoint freedom from loco-regional relapse (ffLRR). Survival time and time to loco-regional relapse were determined as time point from start of radiotherapy till time of death or loco-regional recurrence, or last follow-up.

### Statistical analysis

#### Classifier building and leave-one-out cross-validation

A prognostic six-parameter classifier was built from the genotypes of eight SNPs in seven candidate genes associated with base excision, nucleotide excision, and DNA double strand break repair along with standard clinical covariates. A score selection method for proportional hazard regression with leave-one-out cross-validation (LOO-CV) was used to assure internal validity [22, 23]. The six-parameter model with the highest score *χ*^2^ statistic prevalent in more than 60% of the leave-one-out training datasets was further evaluated. The LOO-CV approach was performed using the SAS macro described by Rushing et al. [24]. The PHREG and LIFETEST procedures were used from SAS version 9.4 [22]. Model selection and classifier calibration was performed on a training dataset, leaving each time the *i*-th patient out. The *i*-th patient was than classified as high or low risk depending on its predictive risk score according to the classifier from the training dataset. This procedure was repeated for each patient. The maximum number of parameters included into the classifier built on the training dataset was limited to six variables. The procedure PHREG selects under the score option the subset of six variables with the highest likelihood score statistic. If less than six parameters were selected in more than 60% of the training datasets, the number of covariates included into the classifier was reduced to the number of covariates fulfilling the former criterion. Patients with covariates leading to a higher than the median hazard in the training dataset, were classified as high risk. This procedure was repeated for patient *i* = 1 to *N* (*N* = number of patients in this study). Kaplan–Meier survivor functions in the high and low-risk groups were compared with a log-rank test, the Wilcoxon test. In addition, survival and loco-regional recurrence analysis was performed using the proportional hazard analysis. The validity of the proportional hazards assumption was assessed by a Kolmogorov-type supremum test (procedure PHREG from SAS).

The Hardy–Weinberg equilibrium of the alleles at an SNP position was analysed using a goodness-of-fit *χ*^2^ test [23]. Linkage disequilibrium between genotype distributions of two SNPs was characterized by the Pearson’s correlation coefficient from a 3 × 3 contingency table [25]. The strength of the correlation between genotypes was tested by Fisher’s exact test (procedure FREQ, SAS).

## Results

Characteristics of the eligible patients from the DKTK-ROG biomarker study involving a total of 158 patients with complete clinical data for multivariable analyses and DNA available for SNP-genotyping are shown in Table 1a. A set of 132 patients formed the basis of the present study (exclusion due to unavailability of DNA biomaterial). In eight patients without p16 immunohistochemical staining results, the average prognostic p16-effect was considered by a p16-dummy variable. The total radiation doses applied ranged from 68.4 to 74.0 Gy, median 72.0 Gy. Endpoints for this biomarker study were 5-year-overall survival as the first and freedom from loco-regional progression as the second endpoint. A total of 65 patients died during follow-up and 53 had a documented loco-regional recurrence. A group of 20 patients was separately documented by the DKTK-ROG. Patients who had received cisplatin-based induction chemotherapy followed by concurrent cisplatin-based definitive radiochemotherapy for squamous cell head and neck carcinoma during the same time period were available as a validation group for the prognostic survival risk model. Their characteristics are shown in Table 1b. The total radiation doses applied ranged from 70 to 72 Gy in this group of patients. Eleven patients died during follow-up, while only five experienced a loco-regional recurrence. This event number was too low for validation of the loco-regional relapse endpoint.

**Table 1:** (a) Patient characteristics and clinical prognostic factors of patients from the DKTK-ROG definitive radiotherapy biomarker study with genotyping. (b) Validation group for the survival risk factor model. Patients received cisplatin-based induction chemotherapy plus definitive radiotherapy and concurrent chemotherapy and were registered by the DKTK-ROG.

(a)	
Gender (female, male)	23/109
Age (median, range) [years]	58.1 (39.2–81.9)
Lymph node category (*N*0, *N*1, *N*2, *N*3)	24/7/95/6
*T* stage (*T*2, *T*3, *T*4)	16/34/82
UICC Stage (III, IV)	12/120
Concurrent chemotherapy (cisplatin-based, mitomycin C-based)	108/24
p16 expression (negative, positive, missing)	106/18/8
GTV_total_ (median, range) [cm^3^]	37.3 (5.6–221.8)
Tumor site (oral cavity, oropharynx, hypopharynx)	24/66/42
(b)	
Gender (female, male)	1/19
Age (median, range) [years]	55.2 (41.0–72.7)
N stage (*N*0, *N*1, *N*2, *N*3)	1/8/11/0
UICC Stage (III, IV)	0/20
Concurrent chemotherapy (cisplatin-based, mitomycin C-based)	20/0
p16 expression (negative, positive, missing)	18/2/0
GTV_total_ (median, range) [cm^3^]	38.3 (8.1–204.0)

All numbers represent patient counts, except the rows headed by age and GTV_total_. The 7th edition of the UICC classification was applied.

*GTV*_*total*_ combined gross tumor volume of the primary tumor and positive lymph nodes.

The allele frequencies of the evaluated candidate SNPs are shown in Table 2 for the 132 patients from the DKTK-ROG biomarker study. No deviations from Hardy–Weinberg equilibrium were observed for all eight SNPs (Table 2). The genotype of rs13181 was correlated with rs1799793. The Pearson’s correlation coefficient was 0.68 for 95% confidence interval (95% CI: 0.59–0.79), *p* < 0.0001, Fisher’s exact test. This genotype correlation based on the correlation of the rs1799793-1 and rs13181-1 contrast variables (*r*_Pearson_ = 0.70), while rs1799793-1 and rs13181-2 showed only a slight correlation (*r*_Pearson_ = 0.29). Homozygote major alleles at both loci were observed in 40 patients, heterozygote alleles in 46, and homozygote minor at both loci in 12 patients. There were no notable correlations between the alleles of the other pairs of SNPs (with absolute values of *r*_s_ < 0.20) except for the pair rs11615 and rs1799793 (*r*_Pearson_ = 0.36 (95% CI: 0.23–0.53), *p* < 0.0001, Fisher’s exact test).

**Table 2:** SNP genotype frequencies in the DKTK-ROG definitive radiotherapy dataset.

SNP	Gene	Genotypes, *n* (%)			*p* value assuming Hardy–Weinberg equilibrium
rs1799793	*ERCC2*	GG: 48 (36.4%)	GA: 65 (49.2%)	AA: 19 (14.4%)	0.73
rs13181	*ERCC2*	AA: 49 (37.1%)	CA: 60 (45.5%)	CC: 23 (17.4%)	0.53
rs50871	*ERCC2*	AA: 45 (34.1%)	AC: 63 (47.7%)	CC: 24 (18.2%)	0.81
rs2267437	*XRCC6*	CC: 39 (29.6%)	CG: 65 (49.2%)	GG: 28 (21.2%)	0.92
rs11615	*ERCC1*	TT: 62 (47.0%)	TC: 58 (43.9%)	CC: 12 (9.1%)	0.77
rs4988023	*ATM*	AA: 98 (74.2%)	AC: 29 (22.0%)	CC: 5 (3.8%)	0.14
rs17655	*ERCC5*	GG: 80 (60.6%)	CG: 47 (35.6%)	CC: 5 (3.8%)	0.55
rs25487	*XRCC1*	GG: 52 (39.4%)	GA: 66 (50.0%)	AA: 14 (10.6%)	0.30

**Fig. 1:**
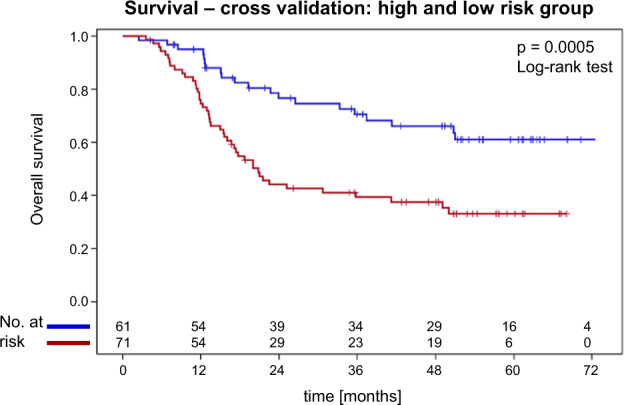
Survival—cross-validation: high and low-risk group. Cross-validated survival curves in the high (red) and low-risk (blue) groups separated at the median prognostic index from the trainings datasets entering the SNP genotype data in addition to the clinical covariates into the model. There was a significant difference between curves (*p* = 0.0005, log-rank test).

For the identification of clinical or SNP markers associated with survival or loco-regional recurrences, a score selection method for proportional hazard regression with LOO-CV was used [22, 23]. The identified six markers for overall survival and freedom from loco-regional recurrence are shown in Table 3. The Fig. 1 shows the cross-validated Kaplan–Meier survival curves according to this six-parameter classifier, that was highly predictive (*p* < 0.0005, log-rank test).

**Table 3:** Markers identified by best six covariate subset score selection proportional hazard analysis and leave-one-out cross-validation.

Endpoint	Covariate 1 (prevalence in LOO training sets)	Covariate 2 (prevalence in LOO training sets)	Covariate 3 (prevalence in LOO training sets)	Covariate 4 (prevalence in LOO training sets)	Covariate 5 (prevalence in LOO training sets)	Covariate 6 (prevalence in LOO training sets)
Survival	log(GTV_total_) (100%)	Type-concurrCTX (100%)	rs1799793-1 (87%)	rs13181-2 (98%)	P16 (98%)	P16-dummy-var (84%)
Loco-regional control	log(GTV_total_) (100%)	rs17655-1 (100%)	rs13181-2 (99%)	rs17655-2 (92%)	rs1799793-1 (95%)	rs1799793-2 (92%)

*LOO* leave-one-out, *log(GTV*_*total*_*)* logarithm to the base e of the combined gross tumor volume of the primary tumor and positive lymph nodes, *Type-concurrCTX* type of concurrent chemotherapy given (cisplatin-based vs. mitomycin C-based), *p16* p16 over-expression (positive vs. negative), *p16-dummy-var* dummy variable indicating whether p16 immunohistochemistry is available or missing.

**Fig. 2:**
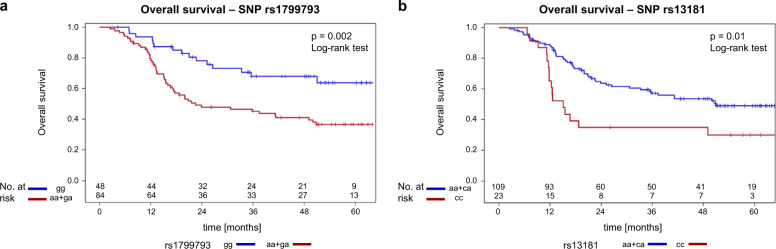
Overall survival: SNP rs1799793 and SNP rs13181. **a** Overall survival—SNP rs1799793. Overall survival curves of the patients included in this study according to rs1799793 GG-major vs. AA + GA genotypes (*p* = 0.002, log-rank test). **b** Overall survival—SNP rs13181. Survival curves of the patients according to rs13181 genotypes.

The six identified markers were analyzed in detail using univariate proportional hazard analysis. From the clinical covariates the logarithm of the total gross tumor volume und p16 were related to survival at a *p* value < 0.05. Two SNPs were associated with survival, rs1799793-1 (*p* = 0.0031) and rs13181-2 (*p* = 0.017). The hazard ratio (HR) for survival according to rs1799793 was 0.418 (95% CI: 0.234–0.744) comparing the major GG genotype with the pooled AA or GA genotypes (Fig. 2a). The rs13181 homozygote minor genotype CC was associated with an HR of 2.074 (95% CI: 1.177–3.658) for survival in comparison to AC as well as AA and identified a subgroup of 17% of patients with a worse prognosis. The survival curves according to rs13181-2 are depicted in Fig. 2b. Multivariable analysis using forward selection from the identified six markers revealed that log(GTV_total_), rs1799793-1, and the type of concurrent chemotherapy, cisplatin vs. mitomycin C were simultaneously correlated with survival at *p* < 0.05 (Table 5).

The association of the SNP markers rs1799793-1 and rs13181-2 with survival was also analyzed in the validation dataset of 20 patients receiving induction chemotherapy. The rs13181-2 was associated with survival (*p* = 0.030, score *χ*^2^ test) and the HR was 9.0 for the homozygote minor patients vs. the others. There was a trend toward longer survival in rs1799793 homozygote major patients vs. the others (HR: 0.378, *p* = 0.089, score *χ*^2^ test). Because of the small sample size, this validation has to be regarded as preliminary. A prospective biomarker validation study is underway by the DKTK-ROG and has finished patient recruitment in 2018.

**Fig. 3:**
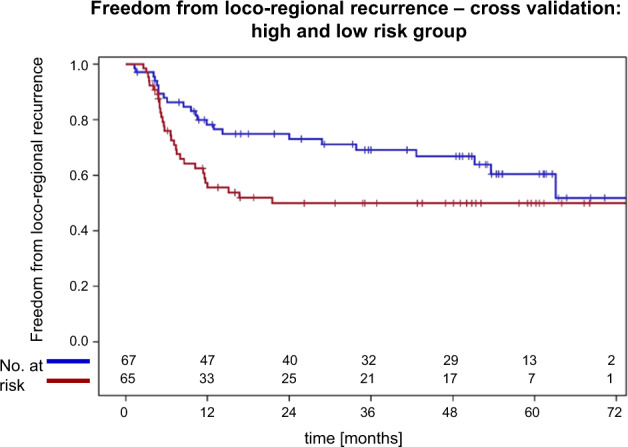
Freedom from loco-regional recurrence—cross-validation: high and low-risk group. Cross-validated freedom from loco-regional recurrence curves in the high (red) and low-risk group (blue) defined by SNP genotypes and clinical covariates. The log-rank and the Wilcoxon test for comparison of these curves resulted in *p* = 0.062 and *p* = 0.025, respectively.

**Fig. 4:**
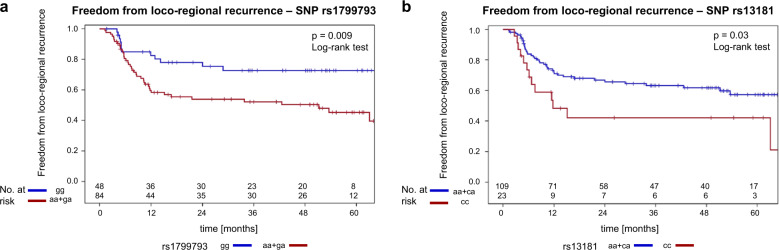
Freedom from loco-regional recurrence: SNP rs1799793 and SNP rs13181. **a** Freedom from loco-regional recurrence—SNP rs1799793. Freedom from loco-regional recurrence curves according to rs1799793 genotypes (*p* = 0.009, log-rank test). **b** Freedom from loco-regional recurrence—SNP rs13181. Freedom from loco-regional recurrence curves according to rs13181 genotypes (*p* = 0.03, log-rank test).

The six-parameter model with the highest score *χ*^2^ statistic for association with freedom from loco-regional recurrence is shown in Table 3. The cross-validated freedom from loco-regional recurrences curves of the high and low-risk groups are shown in Fig. 3. The *p* value for comparison of these curves was *p* = 0.062 using the log-rank test and *p* = 0.025 using the Wilcoxon test. As both curves do not show an increasing divergence with follow-up, deviations from the proportional hazards assumptions were suspected, but not detected by the Kolmogorov–Smirnov type supremum test (*p* = 0.22). In that case, the Wilcoxon test can have a larger power than the log-rank test [26]. The results of univariate proportional hazard analysis of all selected markers are shown in Table 4. At the freedom from loco-regional recurrence endpoint the log(GTV_total_), rs1799793-1, and rs13181-2 were associated with a *p* value of <0.05. Freedom from loco-regional recurrence curves according to rs1799793-1, and rs13181-2 are shown in Fig. 4a, b. Due to the correlation between rs1799793-1 and rs13181-2, multivariable analysis selected log(GTV_total_), rs1799793-1, and rs17655-1 as independent prognostic factors by the forward method (Table 5).

**Table 4:** Univariate proportional hazard analysis of the clinical and SNP genotype covariates selected by the cross-validated best six covariate subset score selection procedure.

	Hazard ratio (95% CI)	*p* value
Predictive covariate for survival		
log(GTV_total_) [cm^3^]	2.092 (1.496–2.927)	<0.0001
rs1799793-1 (hz-major vs. other)	0.418 (0.234–0.744)	0.0031
rs13181-2 (hz-minor vs. other)	2.074 (1.177–3.658)	0.017
p16expr. (pos vs. neg)	0.369 (0.248–0.922)	0.033
Type-concurrCTX (MMC vs. cisplatin)	0.497 (0.227–1.089)	0.081
p16-dummy-var (nd vs. neg)	0.381 (0.093–1.564)	0.18
Predictive covariate for LRC		
log(GTV_total_) [cm^3^]	1.914 (1.317–2.780)	0.0007
rs1799793-1 (hz-major vs. other)	0.435 (0.228–0.829)	0.011
rs13181-2 (hz-minor vs. other)	1.974 (1.054–3.699)	0.034
rs17655-1 (hz-major vs. other)	0.630 (0.368–1.080)	0.093
rs17655-2 (hz-minor vs. other)	0.365 (0.050–2.638)	0.32
rs1799793-2 (hz-minor vs. other)	1.247 (0.587–2.647)	0.57

*LOO* leave-one-out, *log(GTV*_*total*_*)* logarithm to the base *e* of the combined gross tumor volume of the primary tumor and positive lymph nodes, *Type-concurrCTX* type of concurrent chemotherapy given (cisplatin-based vs. mitomycin C-based), *p16* p16 over-expression (positive vs. negative), *p16-dummy-var* dummy variable indicating whether p16 immunohistochemistry is available or missing.

**Table 5:** Proportional hazard multivariable analysis of the clinical and SNP genotype covariates identifies the best six covariate subset score selection procedure using forward selection.

	Hazard ratio (95% CI)	*p* value
Predictive covariate for survival		
log(GTV_total_) [cm^3^]	2.117 (1.552–3.053)	<0.0001
rs1799793-1 (hz-major vs. other)	0.416 (0.233–0.745)	0.0032
Type-concurrCTX (MMC vs. cisplatin)	0.411 (0.186–0.909)	0.028
Predictive covariate for LRC		
log(GTV_total_) [cm^3^]	1.980 (1.344–2.918)	0.0006
rs1799793-1 (hz-major vs. other)	0.480 (0.251–0.918)	0.027
rs17655-1 (hz-major vs. other)	0.560 (0.325–0.968)	0.038

*LOO* leave-one-out, *log(GTV*_*total*_*)* logarithm to the base e of the combined gross tumor volume of the primary tumor and positive lymph nodes, *Type-concurrCTX* type of concurrent chemotherapy given (cisplatin-based vs. mitomycin C-based), *p16* p16 over-expression (positive vs. negative), *p16-dummy-var* dummy variable indicating whether p16 immunohistochemistry is available or missing.

## Discussion

This retrospective multicentre study analyzed the predictive value of SNPs in genes associated with nucleotide excision repair, which is a major repair pathway for removal cisplatin–DNA or mitomycin C adducts like *ERCC2*, *ERCC1* and *ERCC5*. In addition, SNPs on DNA single (*XRCC1*) and double strand break repair genes (*XRCC6, ATM*) were analyzed. Patients with locally advanced head and neck cancer were treated with definitive radiotherapy and concurrent chemotherapy.

In this study, the homozygote major GG rs1799793 genotype was associated with improved and the homozygote minor CC rs13181 genotype with worse survival or ffLRR than other respective genotypes in patients with locally advanced oro- or hypopharyngeal or oral cavity carcinoma, treated with concurrent radiochemotherapy. The rs1799793 minor allele frequency with 39% in this study is similar to that in other European samples, as obtained from HaploReg v.4.1 data [27]. The observations made by Lopes-Aguiar [28] were heading in the same direction, using a dominant model for rs1799793 after concurrent definitive radiochemotherapy in a smaller group of patients. Farnebo et al. [7] also found worse survival in homozygote minor allele rs13181 patients after definitive radiotherapy for head and neck carcinoma using a recessive model. In stages I and II of head and neck cancer treated with radiotherapy alone, no significant prognostic value of rs1799793 or rs13181 on overall survival was found in two studies [2, 17]. Other retrospective studies on the prognostic value of *ERCC2* SNPs enrolled heterogeneously treated patients, including those treated with surgery [11, 12] or did not use overall survival as an endpoint [11]. The study by Zhong et al. [12] on patients treated with surgery with or without postoperative radiotherapy [29] concluded that a prognostic effect of rs13181 might be therapy dependent [12].

Mechanisms which could explain a decreased effectiveness of cisplatin-based chemotherapy and radiotherapy in patients with rs13181 and rs1799793 variant-type tumor cells are: (1) stronger synchronization in the S-phase due to intensely induced p53 expression [30] during fractionated irradiation or (2) less chromosomal damage after X-rays in minor type rs13181 cells [16]. The minor variant of rs1799793 is associated with reduced mRNA levels [31, 32]. Moisan et al. found that reduced expression of *ERCC2* RNA can lead to a G2/M block and thereby alter radiation sensitivity of cycling cells [33]. In addition, variants of the *ERCC2* gene at codons 312 and 751 might alter the mutational spectrum of tumors in these patients and thereby modify the sensitivity toward radiochemotherapy [34]. In nonsmall-cell lung cancer lower response rates to palliative cisplatin-based chemotherapy were found in rs13181 [35] and rs1799793 minor variants [36] using a recessive model in concordance with the findings of the present study.

The DNA for genotyping of the SNPs analyzed in this trial was obtained from FFPE-tumor probes containing various amounts of normal and tumor tissues in contrast to peripheral blood lymphocytes in most other studies. However, a total of 99% concordance rate for SNP in *ERCC2* genotyping from FFPE colorectal tumor material and peripheral blood was found in the study of Van Huis-Tanja [37]. In addition, somatic mutations in tumors in the *ERCC2* gene never affected the rs13181 or rs1799793 site [38]. Therefore, the results from both sources of cells are likely to be in concordance with one another.

The internal validity of *ERCC2* SNPs as prognostic factors was analyzed by LOO-CV. The external validity was analyzed using data from patients receiving induction chemotherapy and cisplatin-based concurrent radiochemotherapy and will be further analysed in a prospective multicentre trial of the DKTK-ROG [14, 15].

The rs1799793 and rs13181 SNPs at *ERCC2* had a high predictive value for overall survival and freedom from loco-regional recurrence after definitive radiochemotherapy. Predictive tools are urgently needed for radiation dose escalation or further treatment intensification for high risk patients receiving cisplatin-based radiochemotherapy so long as long-term prognosis of these patients is below or about 50%. While this study is larger, and the group of selected patients more homogeneously treated in comparison to previous studies evaluating the interference of *ERCC2* SNPs with outcome, further validation is warranted. A prospective biomarker study of the DKTK-ROG is underway for validation and enforcement of the clinical relevance of our findings.
