# Correction: *ERCC2* gene single-nucleotide polymorphism as a prognostic factor for locally advanced head and neck carcinomas after definitive cisplatin-based radiochemotherapy

**DOI:** 10.1038/s41397-020-00193-6

**Published:** 2020-10-02

**Authors:** Maja Guberina, Ali Sak, Christoph Pöttgen, Ingeborg Tinhofer-Keilholz, Volker Budach, Panagiotis Balermpas, Jens Von der Grün, Claus Michael Rödel, Eleni Gkika, Anca-Ligia Grosu, Amir Abdollahi, Jürgen Debus, Claus Belka, Steffi Pigorsch, Stephanie E. Combs, David Mönnich, Daniel Zips, Chiara De-Colle, Stefan Welz, Annett Linge, Fabian Lohaus, Gustavo Baretton, Thomas Gauler, Michael Baumann, Mechthild Krause, Martin Schuler, Agnes Bankfalvi, Benedikt Höing, Stephan Lang, Martin Stuschke

**Affiliations:** 1 Department of Radiotherapy, University Hospital Essen, University of Duisburg-Essen, Essen, Germany; 2 German Cancer Consortium (DKTK), Partner Site Berlin, and German Cancer Research Center (DKFZ), Heidelberg, Germany; 3 Department of Radiooncology and Radiotherapy, Charité—Universitätsmedizin Berlin, Corporate Member of Freie Universität Berlin, Humboldt-Universität zu Berlin, and Berlin Institute of Health, Berlin, Germany; 4 German Cancer Consortium (DKTK), Partner Site Frankfurt, and German Cancer Research Center (DKFZ), Heidelberg, Germany; 5 Department of Radiotherapy and Oncology, Goethe-University Frankfurt, Frankfurt, Germany; 6 Department of Radiation Oncology, Medical Center, Medical Faculty, University of Freiburg, Freiburg, Germany; 7 German Cancer Consortium (DKTK), Partner Site Freiburg, and German Cancer Research Center (DKFZ), Heidelberg, Germany; 8 German Cancer Consortium (DKTK), Partner Site Heidelberg, and German Cancer Research Center (DKFZ), Heidelberg, Germany; 9 Heidelberg Institute of Radiation Oncology (HIRO), National Center for Radiation Research in Oncology (NCRO), University of Heidelberg Medical School, and German Cancer Research Center (DKFZ), Heidelberg, Germany; 10 Department of Radiation Oncology, Heidelberg Ion Therapy Center (HIT), University of Heidelberg Medical School, Heidelberg, Germany; 11 National Center for Tumor Diseases (NCT), Medicine and University Hospital, Technische Universität Dresden, Partner Site Dresden, Germany and German Cancer Research Center (DKFZ), Heidelberg, Germany; 12 Translational Radiation Oncology, University of Heidelberg Medical School, and German Cancer Research Center (DKFZ), Heidelberg, Germany; 13 Clinical Cooperation Unit Radiation Oncology, University of Heidelberg Medical School and German Cancer Research Center (DKF), Heidelberg, Germany; 14 German Cancer Consortium (DKTK), Partner Site Munich, and German Cancer Research Center (DKFZ), Heidelberg, Germany; 15 Department of Radiotherapy and Radiation Oncology, University Hospital, Ludwig-Maximilians-Universität, Munich, Germany; 16 Clinical Cooperation Group Personalized Radiotherapy in Head and Neck Cancer, Helmholtz Zentrum Munich, Neuherberg, Germany; 17 Department of Radiation Oncology, Technische Universität München, Munich, Germany; 18 Department of Radiation Sciences (DRS), Institut für Innovative Radiotherapie (iRT), Helmholtz Zentrum Munich, Neuherberg, Germany; 19 German Cancer Consortium (DKTK), Partner Site Tübingen, and German Cancer Research Center (DKFZ), Heidelberg, Germany; 20 Department of Radiation Oncology, Faculty of Medicine and University Hospital Tübingen, Eberhard Karls Universität Tübingen, Tübingen, Germany; 21 OncoRay—National Center for Radiation Research in Oncology, Faculty of Medicine and University Hospital Carl Gustav Carus, Technische Universität Dresden, Helmholtz-Zentrum Dresden—Rossendorf, Dresden, Germany; 22 Department of Radiotherapy and Radiation Oncology, Faculty of Medicine and University Hospital Carl Gustav Carus, Dresden, Technische Universität Dresden, Dresden, Germany; 23 German Cancer Consortium (DKTK), Partner Site Dresden, and German Cancer Research Center (DKFZ), Heidelberg, Germany; 24 Helmholtz Association/Helmholtz-Zentrum Dresden—Rossendorf (HZDR), Dresden, Germany; 25 German Cancer Research Center (DKFZ), Heidelberg, Germany; 26 Tumor and Normal Tissue Bank, University Cancer Centre (UCC), University Hospital Carl Gustav Carus, Technische Universität Dresden, Dresden, Germany; 27 Helmholtz-Zentrum Dresden—Rossendorf, Institute of Radiooncology—OncoRay, Dresden, Germany; 28 German Cancer Consortium (DKTK), Partner Site University Hospital Essen, and German Cancer Research Center (DKFZ), Essen, Germany; 29 Department of Medical Oncology, West German Cancer Center, University Hospital Essen, University Duisburg-Essen, Hufelandstrasse 55, Essen, Germany; 30 Division of Thoracic Oncology, University Medicine Essen-Ruhrlandklinik, University Duisburg-Essen, Essen, Germany; 31 Institute for Pathology, University Hospital Essen, University of Duisburg-Essen, Essen, Germany; 32 Department of Otorhinolaryngology, University Hospital Essen, University Hospital Duisburg-Essen, Essen, Germany

**Keywords:** Cancer genetics, Prognostic markers

Correction to: *The Pharmacogenomics Journal*

10.1038/s41397-020-0174-1

The original version of this Article contained an error in the spelling of the author Eleni Gkika, which was incorrectly given as Eleni Gikka. As well as the author Stephanie E. Combs, this was incorrectly given as Stephanie Combs. Both have now been corrected in the PDF and HTML versions of the Article.

